# Strangulated Amyand’s hernia containing gangrenous appendix and cecum in a preterm neonate

**DOI:** 10.1093/jscr/rjag193

**Published:** 2026-03-26

**Authors:** Majd Oweidat, Fatima Zain Hanini, Alzahra Akram Hamdan, Layali Jamal Haymoni, Raef Najajra, Mahmmoud A I Sawalha, Anas Ishqair, Ihsan Ghazzawi, Abdelrazzaq Abu Mayaleh

**Affiliations:** College of Medicine, Hebron University, West Bank, Hebron, Palestine; Palestine Red Crescent Specialized Hospital–Hebron, PRCS, West Bank, Hebron, Palestine; College of Medicine, Hebron University, West Bank, Hebron, Palestine; Palestine Red Crescent Specialized Hospital–Hebron, PRCS, West Bank, Hebron, Palestine; College of Medicine, Hebron University, West Bank, Hebron, Palestine; Palestine Red Crescent Specialized Hospital–Hebron, PRCS, West Bank, Hebron, Palestine; College of Medicine, Hebron University, West Bank, Hebron, Palestine; Palestine Red Crescent Specialized Hospital–Hebron, PRCS, West Bank, Hebron, Palestine; Department of Pediatrics, Palestine Red Crescent Specialized Hospital – Hebron, PRCS, West Bank, Hebron, Palestine; Department of Pediatrics, Palestine Red Crescent Specialized Hospital – Hebron, PRCS, West Bank, Hebron, Palestine; Faculty of Medicine, Al-Quds University, West Bank, Jerusalem, Palestine; Faculty of Medicine, Al-Quds University, West Bank, Jerusalem, Palestine; Department of Pediatric Surgery, Palestine Red Crescent Specialized Hospital–Hebron, PRCS, Hebron, Palestine; Department of Pediatrics, Palestine Red Crescent Specialized Hospital – Hebron, PRCS, West Bank, Hebron, Palestine; Faculty of Medicine, Al-Quds University, West Bank, Jerusalem, Palestine

**Keywords:** Amyand’s hernia, preterm neonate, strangulated hernia, appendix

## Abstract

Amyand’s hernia defined as the presence of the appendix within an inguinal hernia sac, is rare in neonates and can deteriorate quickly. We report a preterm male twin (33 + 6 weeks; 1460 g) presenting at 1 month with 24 hours of progressive right inguinoscrotal swelling, erythema, irritability, and absent stooling. Exam showed a tender, nonreducible, non-transilluminating mass with abdominal distension. X-ray revealed dilated bowel loops; ultrasound suggested an indirect right inguinal hernia containing an inflamed appendix. Urgent inguinal exploration found incarcerated viable cecum and a gangrenous appendix. Appendectomy through the same incision with high sac ligation and posterior wall repair (no mesh) was performed. Recovery was uneventful: feeds resumed, weight gain continued, and the ipsilateral testis remained viable. This Type 2 Amyand’s hernia shows localized vascular compromise of the appendix in preterm infants and supports immediate targeted ultrasound and prompt surgery to avert perforation, sepsis, and gonadal injury.

## Introduction

An inguinal hernia (IH) is defined as the protrusion of a peritoneal sac through a weak point in the lower abdominal wall at the groin. In children, most IH result from a failure of the processus vaginalis to close during testicular descent [1]. The prevalence of IH is approximately 3%–5% in term infants but may approach 30% in extremely premature infants with birth weights under 1000 g [2].

Amyand’s hernia (AH) is defined as the presence of the appendix within an IH sac. In children this entity is rare, with estimates suggesting that an appendix is found in roughly 0.1%–1% of pediatric IHs overall, and true appendicitis or necrosis of the herniated appendix is described in only a very small subset of those cases (≈0.07%–0.13%) [3, 4]. AH diagnosis has classically been made intraoperatively [1].

Herein, we report what is probably the first reported case of a strangulated AH containing cecum and a gangrenous appendix in a preterm neonate.

## Case presentation

A preterm male neonate presented to our department with a one-day history of progressive right inguinoscrotal swelling noted by his parents, associated with inconsolable crying and increasing redness of the scrotum; the swelling had enlarged over that day, prompting medical assessment. According to the caregivers, there had been decreased oral intake and no bowel motion for approximately 24 hours before presentation, but there was no reported vomiting.

The neonate had been delivered by cesarean section (CS) in the early preterm period at 33 + 6 weeks of gestation following abnormal Doppler in utero during a multiple pregnancy. At birth, his weight was 1460 g, he cried immediately, and Apgar scores were 8 and 8 at one and five minutes, respectively. He passed urine and meconium within the first 24 hours of life. After delivery he was admitted to the neonatal intensive care unit (NICU) for 17 days for prematurity. He was discharged from the NICU at a body weight of 1930 g. Family and social history are unremarkable.

**Figure 1:**
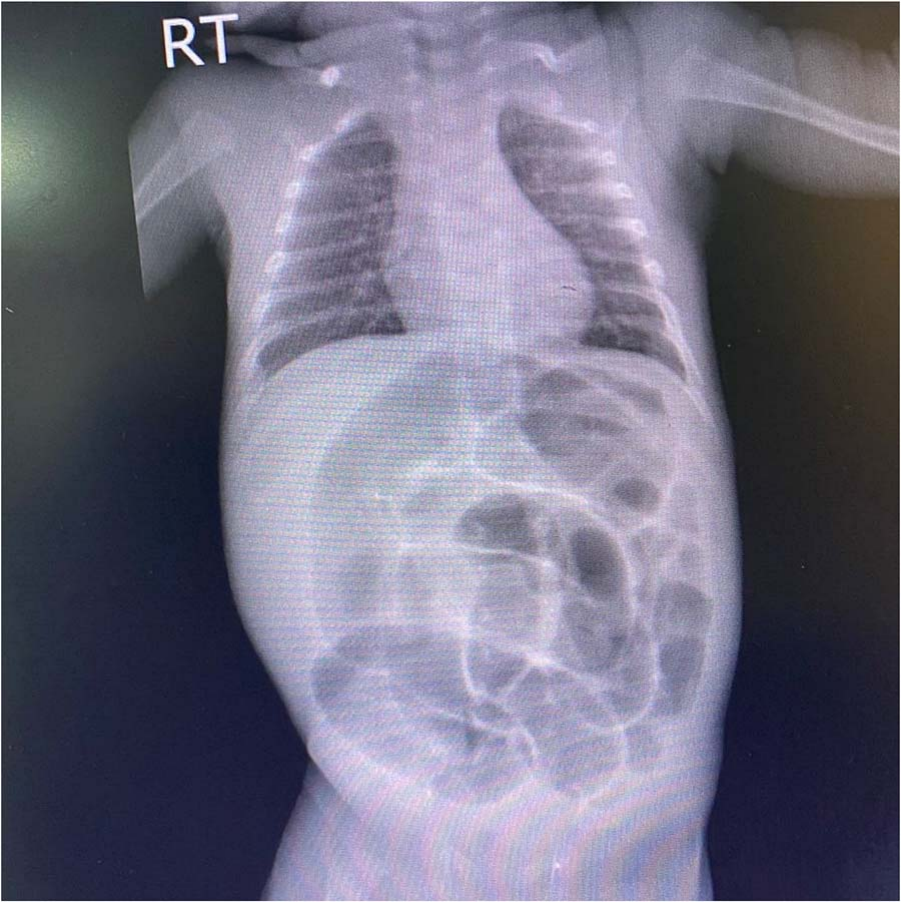
An anteroposterior abdominal X-ray of a male neonate shows marked gaseous distension of the abdomen with severe, diffuse dilation of numerous bowel loops. No evidence of pneumoperitoneum.

On the current presentation, the patient appeared mildly dehydrated but was not in overt distress. Vital signs on admission were temperature 37.9°C, heart rate 176 bpm, respiratory rate 54 bpm, blood pressure 105/55 mmHg, and peripheral oxygen saturation 98%. The patient’s weight is 2265 g (3^rd^ percentile for corrected gestational age), length 42 cm (10th percentile), and head circumference 33 cm (10th percentile). Abdominal examination revealed abdominal distension and generalized abdominal tenderness. Local examination of the groin and scrotum revealed a right inguinoscrotal swelling that was reddish-purple in color, tender, non-reducible, and did not transilluminate. The left testis was normal on palpation. An abdominal X-ray was obtained to evaluate for bowel obstruction and possible perforation, which showed multiple dilated bowel loops with gas patterns (Fig. 1). Ultrasound (US) of the scrotum and groin showed an indirect right IH containing an inflamed appendix, consistent with Amyand hernia. Other systems examinations were unremarkable.

**Figure 2:**
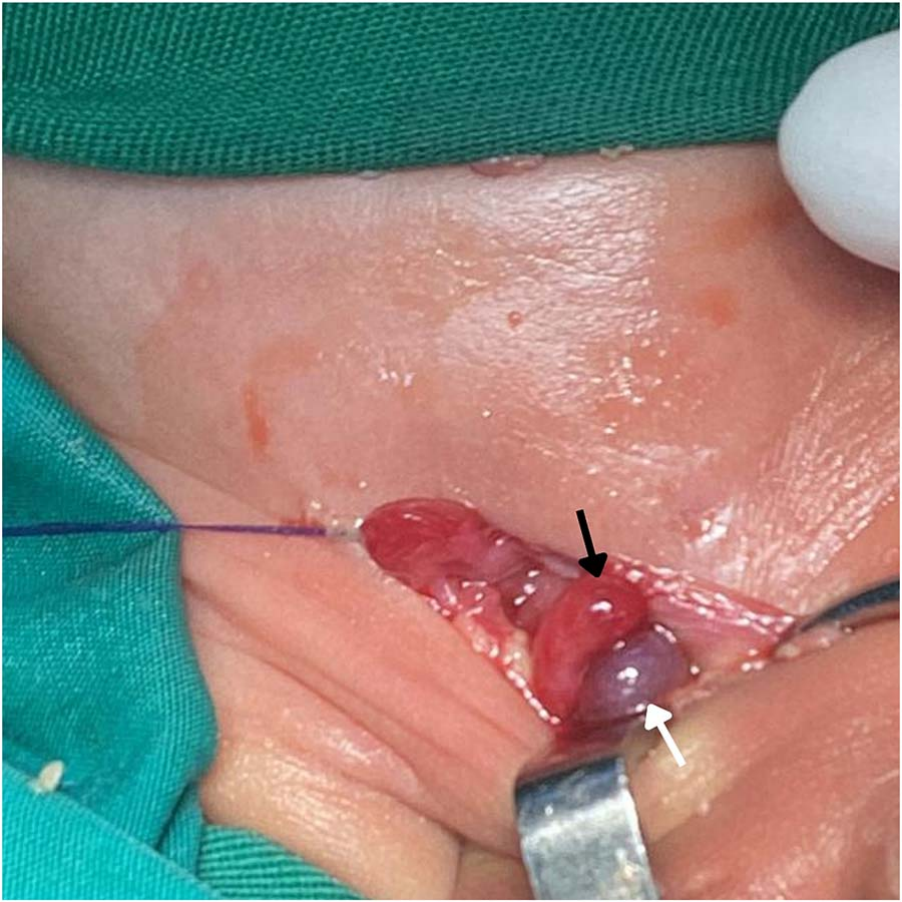
Intraoperative image shows the contents of an AH sac exposed during surgery. The hernia contains the viable cecum (black arrow) and a gangrenous appendix (white arrow).

**Figure 3:**
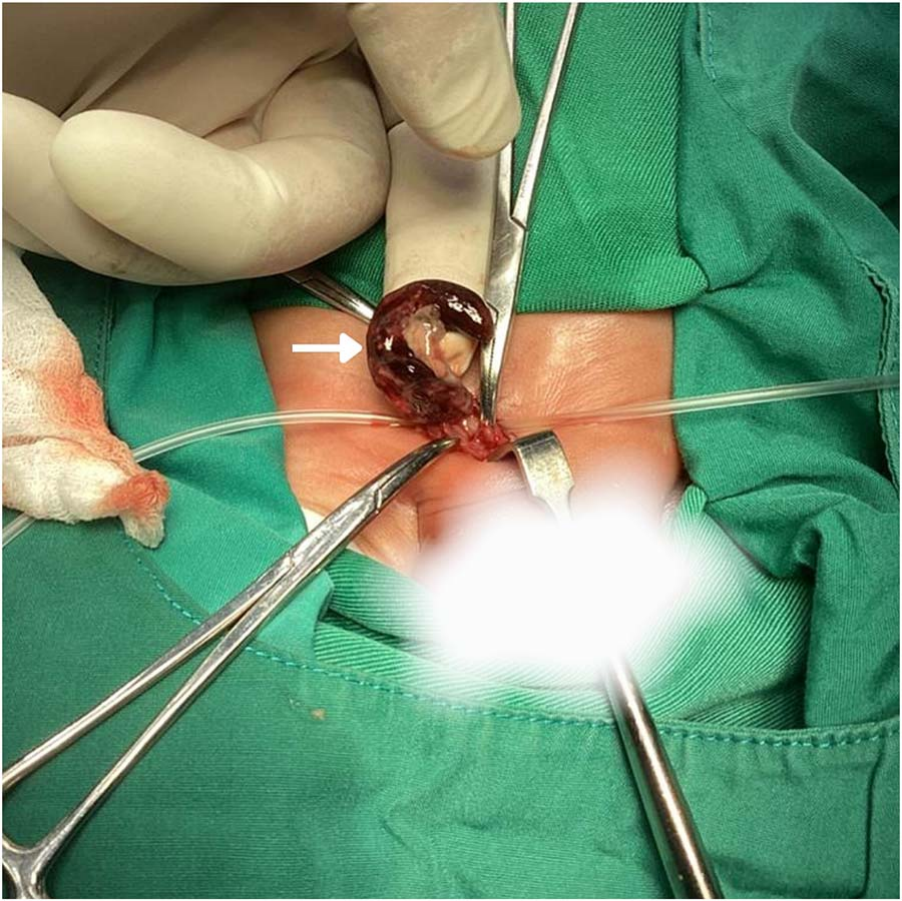
Intraoperative image shows the appendix (white arrow) enlarged and inflamed, with clear areas of gangrenous necrosis.

The neonate was admitted with a diagnosis of a strangulated right IH containing appendix. He was kept nil per os (NPO) and was maintained on intravenous fluids consisting of dextrose 10% prior to operative management. Broad-spectrum intravenous antibiotics were initiated preoperatively, consisting of ampicillin, gentamicin, and metronidazole, with dosing prescribed according to weight-based guidelines. The elevated blood pressure was considered likely secondary to pain, distress, and crying at presentation; repeat measurements after the infant had calmed were within the expected range for age. Under general anesthesia, a right inguinal incision was made. The inguinal canal was opened and the hernial sac was identified and opened (Fig. 2). Sac found to contain both the cecum and the appendix. The cecum was inflamed but viable and the appendix was gangrenous (Fig. 3). The ipsilateral testis appeared slightly dark in color but was viable. The mesoappendix was ligated, and an appendectomy was performed. The hernial sac was released and was then highly ligated. Repair of the posterior wall of the inguinal canal was completed, and the wound was closed in layers. The appendix was sent for histopathological examination, which later demonstrated transmural necrosis with dense neutrophilic infiltration, consistent with acute gangrenous appendicitis.

After surgery, the patient kept NPO with nasogastric tube drainage for 24 hours, then oral feeding gradually resumed and was tolerated. Serial laboratory tests during admission were unremarkable. At the time of discharge, the neonate was afebrile, feeding well, showing weight gain, with a soft abdomen and adequate urine output. Discharge medications included paracetamol prescribed according to weight-based dosing guidelines.

## Discussion

The pathophysiology in this neonate helps explain why AH can deteriorate so fast in neonates. Two mechanisms are described. One suggests that primary appendicitis occurs first, and the already inflamed appendix then herniates. The second, favored in neonates, is vascular: the appendix becomes tightly incarcerated at the internal ring, venous outflow is compromised, and progressive congestion, bacterial overgrowth, and thrombosis produce ischemia, gangrene, or perforation without classic generalized peritonitis [3].

In IH, persistent patency of the processus vaginalis, episodic increases in intra-abdominal pressure, and a relatively lax inguinal canal are recognized contributors. Hernia incarceration and strangulation are a time-critical emergencies [1]. What was unusual in this case was the discovery of both cecum and a necrotic appendix in the sac at such an early age.

Diagnosing AH preoperatively in neonates is challenging because the clinical picture overlaps with other acute scrotal and groin emergencies. Fever and generalized abdominal tenderness may be minimal or absent even when the appendix is ischemic. Historically, most pediatric AH were recognized only intraoperatively [5].

Operative decision-making in AH is commonly guided by the Losanoff and Basson classification, which stratifies cases according to the condition of the appendix and the extent of contamination [6, 7]. This case corresponds to a Type 2 AH. For this category, the recommended approach is appendectomy [6]. The use of prosthetic mesh is discouraged [7]. Testicular preservation is important because complicated neonatal AH has been associated with severe scrotal sepsis, pyocele, and even gonadal infarction, particularly when decompression is delayed [8, 9].

## Conclusion

AH can contain cecum as well as appendix, even in preterm neonates, which can progress quickly to ischemia.
